# An exploratory assessment of stretch-induced transmural myocardial fiber kinematics in right ventricular pressure overload

**DOI:** 10.1038/s41598-021-83154-8

**Published:** 2021-02-11

**Authors:** Danial Sharifi Kia, Ronald Fortunato, Spandan Maiti, Marc A. Simon, Kang Kim

**Affiliations:** 1 Department of Bioengineering, University of Pittsburgh, Pittsburgh, PA USA; 2 Department of Mechanical Engineering and Materials Science, University of Pittsburgh, Pittsburgh, PA USA; 3 Division of Cardiology, Department of Medicine, University of Pittsburgh School of Medicine, 623A Scaife Hall, 3550 Terrace Street, Pittsburgh, PA 15213 USA; 4 Heart and Vascular Institute, University of Pittsburgh Medical Center (UPMC), Pittsburgh, PA USA; 5 Pittsburgh Heart, Lung, Blood and Vascular Medicine Institute, University of Pittsburgh and University of Pittsburgh Medical Center (UPMC), Pittsburgh, PA USA; 6 McGowan Institute for Regenerative Medicine, University of Pittsburgh, Pittsburgh, PA USA; 7 Center for Ultrasound Molecular Imaging and Therapeutics, University of Pittsburgh, Pittsburgh, PA USA

**Keywords:** Heart failure, Hypertension, Cardiomyopathies, Cardiac hypertrophy, Biomedical engineering, Mechanical engineering

## Abstract

Right ventricular (RV) remodeling and longitudinal fiber reorientation in the setting of pulmonary hypertension (PH) affects ventricular structure and function, eventually leading to RV failure. Characterizing the kinematics of myocardial fibers helps better understanding the underlying mechanisms of fiber realignment in PH. In the current work, high-frequency ultrasound imaging and structurally-informed finite element (FE) models were employed for an exploratory evaluation of the stretch-induced kinematics of RV fibers. Image-based experimental evaluation of fiber kinematics in porcine myocardium revealed the capability of affine assumptions to effectively approximate myofiber realignment in the RV free wall. The developed imaging framework provides a noninvasive modality to quantify transmural RV myofiber kinematics in large animal models. FE modeling results demonstrated that chronic pressure overload, but not solely an acute rise in pressures, results in kinematic shift of RV fibers towards the longitudinal direction. Additionally, FE simulations suggest a potential protective role for concentric hypertrophy (increased wall thickness) against fiber reorientation, while eccentric hypertrophy (RV dilation) resulted in longitudinal fiber realignment. Our study improves the current understanding of the role of different remodeling events involved in transmural myofiber reorientation in PH. Future experimentations are warranted to test the model-generated hypotheses.

## Introduction

Transmural myocardial fiber orientations in the right ventricular (RV) wall lead to unique structural and biomechanical properties and play a key role in RV function. Pulmonary hypertension (PH) is a disease that results in RV pressure overload, ventricular remodeling, myocardial hypertrophy and fibrosis. RV failure remains the main cause of mortality in the setting of PH^1^ with 3-year mortality rates as high as 33–38%^2,3^.

Previous studies have closely linked RV biomechanics to ventricular function^4^. From a biomechanical perspective, RV myocardium experiences increased wall thickness, tissue stiffening, fibrosis, chamber dilation and transmural fiber reorientation in PH^5,6^. RV remodeling and fiber reorientation towards the longitudinal (apex to base) direction results in drastically different RV biomechanics, affecting the transmural distribution of wall stress^7^, ventricular twisting motions and filling and ejection mechanics of the RV^8^. Nevertheless, mechanistically, RV fiber reorientation is poorly understood and there remains a debate around the adaptive^9^ vs. maladaptive^8^ nature of fiber remodeling in PH. Characterizing the kinematics of myocardial fibers will help establishing a better understanding of the underlying mechanisms of fiber realignment in PH. However, full-thickness transmural RV fiber kinematics remains largely unexplored, mainly due to imaging difficulties caused by the RV wall thickness in large animal models and human patients, limiting our ability to study fiber kinematics transmurally. Despite high imaging resolutions, the depth limit of optical techniques such as multi-photon microscopy (100–1200 μm limit^10^) and long imaging time, exposure to ionizing radiation, availability and cost-effectiveness issues with diffusion tensor magnetic resonance imaging^11,12^ (DTMRI) limit their applicability for benchtop or clinical studies on RV fiber kinematics. On the other hand, ultrasound imaging is a widely available, non-ionizing, cost-effective technique capable of overcoming the depth limits of optical methods to assess cardiac structure and function.

**Figure 1:**
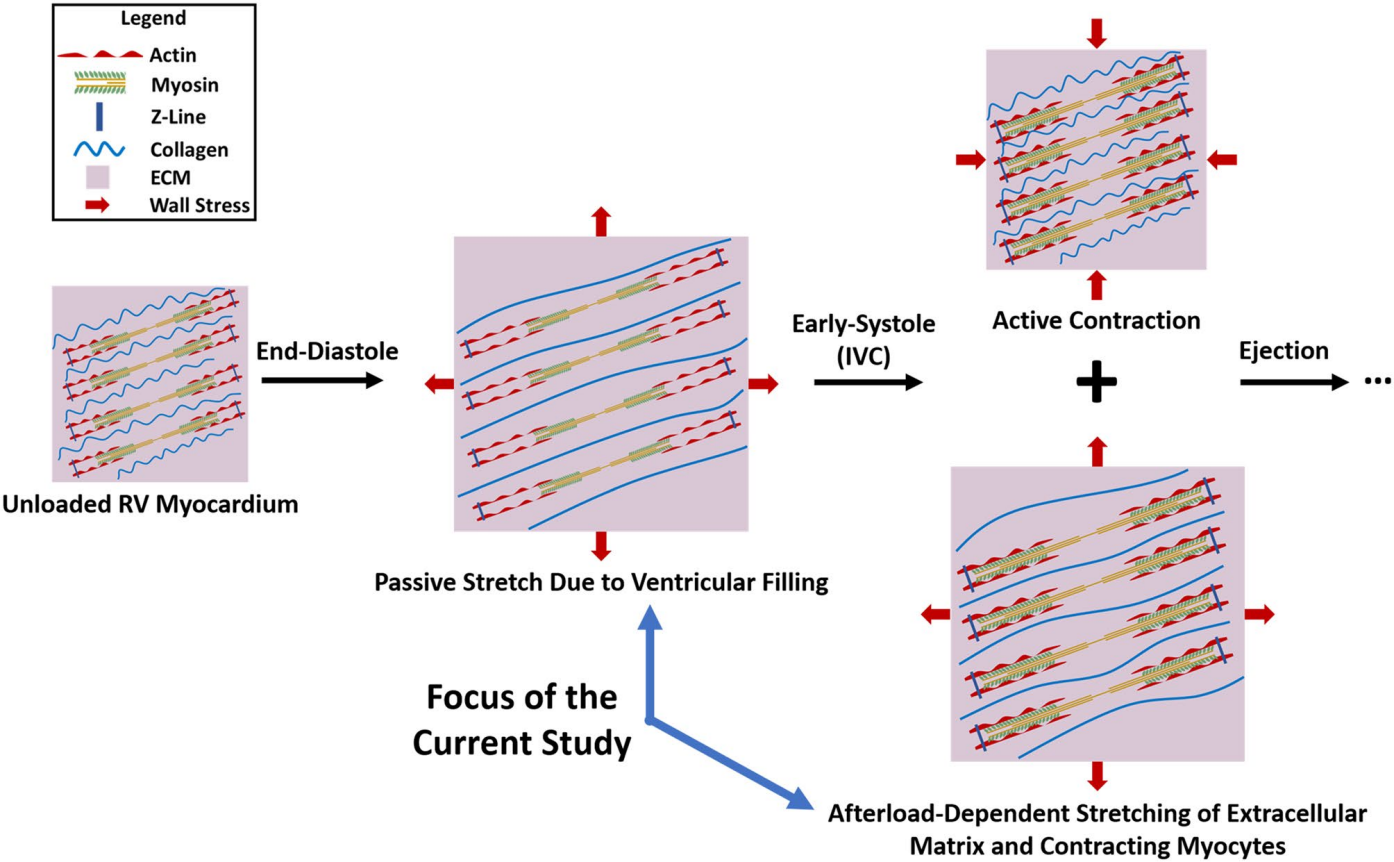
Stretch-induced deformations experienced by RV myocardium during diastolic filling and the IVC phase of early-systole. During diastole, ventricular filling results in passive stretch of the RVFW. IVC follows tricuspid valve closure at end-diastole, during which active myocardial contraction increases chamber pressures (while both tricuspid and pulmonary valves remain closed) and results in increased wall stress (afterload) and stretching of the RVFW. Both active contraction and stretching happen simultaneously during this phase, as reflected in increased ventricular pressures^6,13^ in addition to ventricular wall thinning^13,14^ and fiber stretch^13^ during early-systole. This is followed by pulmonary valve opening and systolic ejection, which results in negative RVFW contractile strains. The current study focuses on stretch-induced deformations of RV myocardium during the end-diastolic and early-systolic phases of a cardiac cycle, without considering the effects of cardiomyocyte contraction. *ECM* extracellular matrix, *RV* right ventricle, *RVFW* right ventricular free wall, *IVC* isovolumetric contraction.

RV myocardium undergoes different modes of deformation during a cardiac cycle (Fig. 1). In the current work we focus on the stretch-induced deformations of right ventricular free wall (RVFW) during diastolic filling and the isovolumic contraction (IVC) phase of early-systole (Fig. 1). IVC follows tricuspid valve closure at end-diastole, when active myocardial contraction increases chamber pressures (while both tricuspid and pulmonary valves remain closed) which results in increased wall stress (afterload) and stretching of the RVFW. Both active contraction and passive stretching happen simultaneously during early-systole, as reflected in increased ventricular pressures^6,13^ (demonstrating active contraction) in addition to fiber stretch^13^ and ventricular wall thinning^13,14^ (demonstrating stretch-induced deformations). This is followed by pulmonary valve opening and systolic ejection. Myocardial contraction (fiber shortening) results in small reversible changes in fiber angles towards the longitudinal direction^15,16^, as also reflected in the torsional motion of the heart^17^. However, longitudinal fiber remodeling in PH has been identified as an end-stage remodeling event^5,6,8^ accompanied by reduced RV ejection fraction^8^ and diminished systolic myofiber shortening^18^ (minimum principle strains). While the eventual outcome of fiber remodeling may affect ventricular contractile mechanics^19,20^, decreased minimum principle strains at end-stage PH^18^ results in smaller myofiber kinematic shifts towards the longitudinal direction compared to normotensive loading and makes myofiber contraction less likely to mechanistically stimulate transmural fiber reorientation in PH. Motivated by this, we hypothesized that stretch-induced deformations experienced by RV myocardium (Fig. 1) in the setting of PH may result in myofiber kinematic shifts towards the longitudinal direction. Stretch-induced deformations have been studied as a stimuli for fiber reorientation in other biological tissues^21,22^.

Stretching of the myocardial niche in early-systole is of particular importance in the setting of pressure overload, mainly due to the increased afterload (wall stress). Previous studies have reported an increase in stretch-activated Ca^2+^ transient amplitudes in response to pressure overload, with no significant alterations in the diastolic sarcomere length^23^. Additionally, expressions of stretch-induced^24^ c-fos proto-oncogenes and fetal isoforms of α-actin have been identified as an early growth-induction response in pressure overload^25^. Increased early-systolic stretch is also evident in echocardiographic assessments of patients with increased RV afterload^26^. Due to material anisotropy and nonlinearity^5,6,27,28^, depending on the ratio of the applied mechanical stimuli (strain), stretching of RV myocardium may lead to fiber kinematics towards different directions under different loading scenarios (supplementary Fig. S1). Stretch-induced alterations in the biomechanical stimuli applied to the myofiber niche has the potential to activate different remodeling pathways leading to fiber realignment and structural remodeling^29–32^.

In the first stage of this study, an enhanced framework was developed and validated against histological measurements to analyze high-frequency ultrasound (HFU) images for quantification of transmural RV myofiber orientations. Stretch-induced myofiber reorientation under uniaxial loading was studied to demonstrate the feasibility of the developed framework to study RV fiber kinematics. Furthermore, due to the complexity associated with coupled biaxial loading/HFU imaging, nonlinear structurally-informed fiber embedded finite element (FE) models of the RV myocardium were developed to conduct an exploratory study on biaxial fiber kinematics. FE models were used to study the role of different remodeling events such as fibrosis^5,6^, concentric^5,6,33^ and eccentric^6,34^ hypertrophy on stretch-induced RV fiber kinematics in acute and chronic pressure overload. Our exploratory study improves the current understanding of the role of different remodeling events involved in transmural reorientation of RV fibers in PH. This will help with appropriate hypothesis generation for future experimental studies on RV fiber kinematics while ongoing work focuses on development of improved imaging algorithms for coupled HFU imaging/biaxial loading.

## Methods

### HFU imaging to quantify transmural RV fiber kinematics

**Figure 2:**
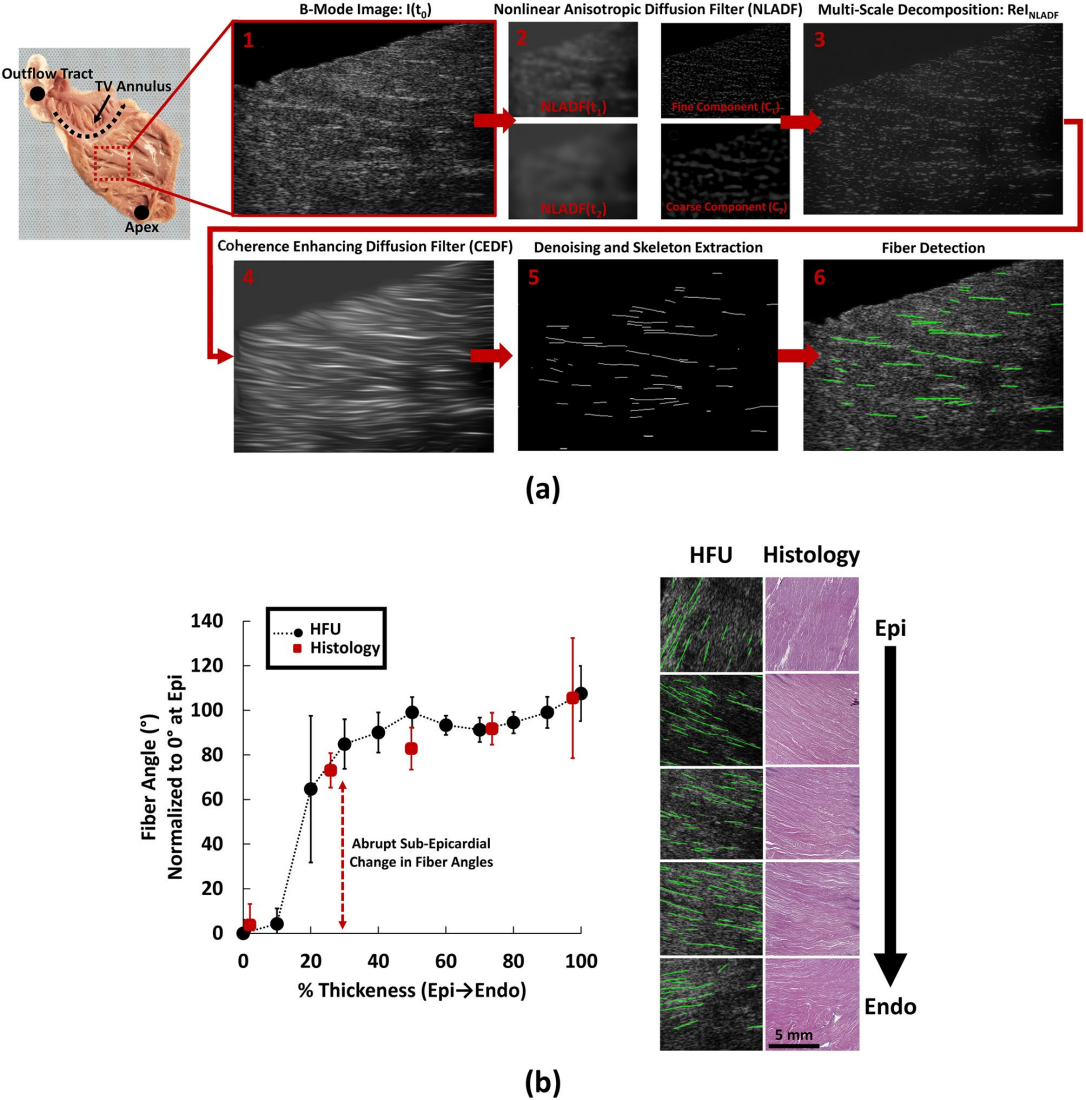
The ultrasound image processing framework used to detect transmural RV myofiber orientations. (**a**) Development of the HFU imaging framework. Basal anterior zone RVFW specimens were scanned while the ultrasound probe was aligned with the apex-to-base direction, normal to the transmural axis. High noise in the acquired high-frequency B-mode images were reduced via multi-scale decomposition using NLADF, followed by establishing myofiber connectivity by CEDF. Skeleton extraction was performed on the resulting image, followed by fiber detection using the Hough transform and fitting a normal PDF to the fiber distributions. (**b**) Algorithm verification by comparing HFU measurements with histological staining (H&E). Error bars demonstrate fiber spread (standard deviation of the distribution of fiber orientations; measure of fiber dispersion) at each transmural section. Fiber angles are normalized to 0° at the epicardial layer, to better demonstrate the transmural change in fiber angles and facilitate comparison with previous studies^42^. Dotted lines between HFU measurements are for visualization purposes only. *TV* Tricuspid valve, *Epi* Epicardium, *Endo* endocardium, *HFU* high-frequency ultrasound, *H&E* hematoxylin and eosin stain, *RV* right ventricle, *RVFW* right ventricular free wall, *PDF* probability density function.

Previous studies have demonstrated the potential of HFU imaging in characterizing muscle fibers with high spatial resolution, using a combination of multi-scale decomposition and diffusion filtering techniques^35,36^. However, to the best of our knowledge, no efforts have been made to quantify the transmural kinematics of RV myofibers using HFU imaging. In the first stage of this study an enhanced HFU imaging framework was developed to study the transmural kinematics of RVFW myofibers (Fig. 2a).

#### Development of an HFU imaging framework

A square specimen was harvested from the basal anterior zone (r2 zone^12^) of porcine RVFW (procured from a local butchery) and scanned at 50 MHz using an HFU scanner (Vevo2100, FUJIFILM-VisualSonics, Toronto, Canada). HFU images were acquired at a precision of 524 pixels/cm while the imaging probe was positioned along the apex to base direction, normal to the transmural axis of the RVFW (from epicardium to endocardium). Using a servo stage, 3D scans were generated by stacks of 2D images acquired at 102 µm increments, chosen based on the transducer elevational beam width. Since HFU scans result in high noise in the acquired images, similar to previous studies^36^, a nonlinear anisotropic diffusion filter (NLADF) was used to reduce the noise levels (Fig. 2a)^37^:

1 $$ \frac{{\partial {\text{I}}}}{{\partial {\text{t}}}} = \nabla .\left( {{\text{c}}\left( {{\text{x}},{\text{y}},{\text{t}}} \right)\nabla {\text{I}}} \right) = {\text{c}}\left( {{\text{x}},{\text{y}},{\text{t}}} \right)\Delta {\text{I}} + \nabla {\text{c }} \cdot { }\nabla {\text{I}} $$

Here, I is the image intensity in 2D space, t represent the iteration steps and c is the anisotropic diffusion tensor at point (x, y), as previously defined^35,36^. The NLADF filtering framework is based on the diffusion equation (directional gaussian blurring^37^) and helps reducing the noise levels while keeping the main features of the image^37^. The acquired B-mode images were filtered using Eq. 1 at two different iterations, NLADF(t_1_) and NLADF(t_2_), while t_2_ > t_1_. Appropriate iteration times (t_1_ and t_2_) were manually selected by observing noise reduction in the denoised images produced by NLADF (t_1_ = 100 and t_2_ = 300 iterations for the purposes of this study). This was followed by multi-scale decomposition of fine and coarse components of the image (Fig. 2a):

2 $$ {\text{C}}_{1} = {\text{ I}}\left( {{\text{t}}_{0} } \right) \, {-}{\text{ NLADF}}\left( {{\text{t}}_{1} } \right) $$

3 $$ {\text{C}}_{2} = {\text{ NLADF}}\left( {{\text{t}}_{1} } \right) \, {-}{\text{ NLADF}}\left( {{\text{t}}_{2} } \right) $$

4 $$ {\text{ReI}}_{{{\text{NLADF}}}} = \upalpha _{1} {\text{C}}_{1} + \upalpha _{2} {\text{C}}_{2} + (1 - \upalpha _{1} - \upalpha _{2} ){\text{NLADF}}({\text{t}}_{2} ) $$

where I(t_0_) is the original B-mode image, C_1_ is the fine component of the HFU image, C_2_ is the coarse component, ReI_NLADF_ is the reconstructed image following NLADF and multi-scale decomposition, and α_1_ and α_2_ are the scaling gains for image reconstruction (Fig. 2a). ReI_NLADF_ helps boosting the fine components in the HFU image, while keeping the main features. Similar to previous work^36^, α_1_ and α_2_ were set at 0.6 and 0.2, respectively.

Despite considerably reduced noise levels after the NLADF, high noise in the original image leads to myofibers represented in interrupted segments (Fig. 2a). Therefore, a coherence enhancing diffusion filter (CEDF)^38^ was used to establish connectivity between disconnected fiber segments, while preserving the orientation of features in the original B-mode image (Fig. 2a). CEDF functions similar to the NLADF algorithm (Eq. 1) while the diffusion tensor c(x,y,t) is replaced by an adaptive structure tensor constructed using localized image orientations^36,38^. This helps completing disconnected segments while keeping the orientation of features in the original image (Fig. 2a). Following each filtering step, the resulting image was denoised via threshold-based techniques^36^. Appropriate denoising thresholds were manually selected for each image.

Finally, the resulting image was converted to a binary format and, subsequent to skeleton extraction, myofiber orientations were detected using the Hough transform (Fig. 2a). A Gaussian probability density function (PDF) was then fitted to the fiber distribution at each section throughout the RV wall to quantify transmural fiber orientations. Distribution means were reported as the dominant orientation at each transmural section, while standard deviation of the distributions represent the fiber spread (dispersion)^39^. Algorithm development was performed using MATLAB (Mathworks, Natick, MA).

#### Algorithm validation with histological measurements

For technical validation purposes, HFU measurements were compared with transmural histological staining of the RVFW specimen using a hematoxylin and eosin (H&E) stain. Fiber orientation of histological sections were detected using gradient based image analysis techniques, as described in our previous studies^5,6^. Briefly, for each histological section, local image gradients were calculated at each pixel, followed by the formation of the second-moment tensor of the gradient map:

5 $$ {\text{H }} = \left[ {\begin{array}{*{20}c} {{\iint }{\text{w}}\left( {{\text{x}},{\text{y}}} \right){\text{I}}_{{\text{x}}} \left( {{\text{x}},{\text{y}}} \right){\text{I}}_{{\text{x}}} \left( {{\text{x}},{\text{y}}} \right){\text{dxdy}}} & {{\iint }{\text{w}}\left( {{\text{x}},{\text{y}}} \right){\text{I}}_{{\text{x}}} \left( {{\text{x}},{\text{y}}} \right){\text{I}}_{{\text{y}}} \left( {{\text{x}},{\text{y}}} \right){\text{dxdy}}} \\ {{\iint }{\text{w}}\left( {{\text{x}},{\text{y}}} \right){\text{I}}_{{\text{x}}} \left( {{\text{x}},{\text{y}}} \right){\text{I}}_{{\text{y}}} \left( {{\text{x}},{\text{y}}} \right){\text{dxdy}}} & {{\iint }{\text{w}}\left( {{\text{x}},{\text{y}}} \right){\text{I}}_{{\text{y}}} \left( {{\text{x}},{\text{y}}} \right){\text{I}}_{{\text{y}}} \left( {{\text{x}},{\text{y}}} \right){\text{dxdy}}} \\ \end{array} } \right] $$

Here, H is the structure tensor (2 × 2 symmetric positive-definite matrix), w(x,y) is a gaussian weighing function specifying the region of interest for integration, and I_x_ and I_y_ are the partial spatial derivatives of the image I(x,y) in x and y directions, respectively (obtained using a cubic B-spline interpolation^40^). Dominant fiber orientations at each transmural section were acquired by evaluating the first eigenvector of the resulting second-moment tensor^40,41^. Image processing and fiber orientation analysis of histological sections were performed using the OrientationJ toolbox^40,41^ in ImageJ (imagej.nih.gov). Similar to previous studies^42^, for algorithm validation and comparison purposes, fiber angles measured via histology and HFU imaging were normalized to 0° at the epicardial layer to better demonstrate the transmural change in fiber angles and eliminate any tissue alignment mismatch between HFU and histological measurements.

#### Feasibility evaluation of the developed framework to study the transmural kinematics of RV myofibers under uniaxial loading

Following algorithm verification, RVFW specimens (n = 3) were harvested from the basal anterior zone of porcine myocardium and loaded using a custom displacement-controlled uniaxial loading gripper that allows real-time HFU imaging. Fiber orientations were measured and analyzed in the RV circumferential-longitudinal coordinate system when apex-to-base denotes the longitudinal direction. Transmural fiber orientations were then analyzed under a stretch ratio (λ) of 1.35 in the circumferential direction. This was chosen based on a RV systolic pressure of ≈ 105 mmHg in PH^34^ and approximation of RVFW stretch based on previously reported biomechanical properties for a porcine model^43^. Experimentally measured fiber kinematics were compared with theoretical predictions for an incompressible transversely isotropic solid with affine kinematics^44^:

6 $$\uptheta _{{{\text{Loaded}}}} = {\text{ arctan}}\left( {\frac{{{\text{Sin}}\left( {\uptheta _{{{\text{Unloaded}}}} } \right){*}\sqrt\uplambda }}{{{\text{Cos}}\left( {\uptheta _{{{\text{Unloaded}}}} } \right){*}\uplambda ^{2} }}} \right) $$

Here, θ_Unloaded_ represents the unloaded fiber orientation at each transmural section, θ_Loaded_ is the fiber angle after loading-induced realignment and λ is the amount of uniaxial stretch. We hypothesized that uniaxial loading results in transmural realignment of RV myofibers towards the loading direction. Additionally, the transmural variation of fiber angles was approximated via linear regression (fiber angles vs. %thickness). Slope of the linear fit to transmural fiber angles demonstrates the helix slope of the RVFW (change in fiber angle per unit thickness), which was hypothesized to decrease in response to uniaxial loading.

### Finite element modeling to study RV fiber kinematics under biaxial loading

While the developed HFU algorithm shows great potential for quantifying RV fiber kinematics, it requires imaging in the plane parallel to the RV wall (normal to the transmural axis). This demands at least one side of the planar RVFW specimen to be free for imaging, which complicates coupled biaxial loading and HFU imaging. Therefore, in the second stage of this study structurally-informed nonlinear-heterogenous-anisotropic FE models of the RVFW were created for an exploratory assessment of biaxial fiber kinematics under different loading scenarios. This will lay the groundwork for future experimental studies on biaxial RV fiber kinematics, while development of specialized loading grippers and improved imaging algorithms for coupled HFU imaging/biaxial loading is in progress.

#### Model development

The modeling techniques used in this study have been described in detail in our previous work^39,45^. Briefly, a custom MATLAB subroutine was used to explicitly create planar fiber networks using the dominant orientation and fiber spreads (pooled standard deviation of orientation distributions from n = 3 samples) measured via HFU imaging. Myofibers, collagen and the ECM were created with 93%, 4% and 3% volume fractions^28,46–48^, respectively, to approximate the tissue-level biaxial properties^43^ of porcine RV myocardium (Table 1). The ECM was modeled using an incompressible isotropic hyperelastic constitutive model^49^:

7 $$ {\text{W}} = \upalpha \left( {{\text{I}}_{{\text{b}}} - 3} \right) +\upbeta \left( {{\text{I}}_{{\text{b}}} - 3} \right)^{2} $$

where α and β are constants representing the ECM mechanical properties, I_b_ is the first invariant of the left Cauchy–Green stretch tensor and W is the strain energy function when J = det(F) = 1 ensures incompressibility (F represents the deformation gradient tensor). Collagen fibers were modeled with linear elastic properties, activated after a recruitment stretch^39^:

8 $$ \sigma = \left\{ {\begin{array}{*{20}l} {0,} \hfill & { \lambda < \lambda_{r} } \hfill \\ {E_{col} \left( {\lambda - \lambda_{r} } \right), } \hfill & { \lambda \ge \lambda_{r} } \hfill \\ \end{array} } \right. $$

Here σ represents components of the Cauchy stress tensor, E_col_ is the stiffness of collagen fibers and λ_r_ is the stretch at which collagen recruitment begins (Table 1). Additionally, myofibers were modeled with a similar approach without a recruitment threshold (Table 1). To find the appropriate model parameters, simulated biaxial stress-stretch response of the generated fiber network was regressed against previously reported material properties of porcine RV myocardium^43^, while goodness of fit was determined using the coefficient of determination (R^2^). For model verification, an unconfined uniaxial loading scenario (under λ = 1.35) was simulated and compared with experimental HFU imaging measurements of RVFW fiber kinematics.

**Table 1:** RV material properties and volume fractions used for FE modeling.

	Volume fraction	Material properties	
ECM (except collagen)	3%^28,46^	α = 0.7 kPa	β = 1.5 kPa
Myofibers	93%	E_myo_ = 10 kPa	
Collagen Fibers	4%^47,48^	E_col_ = 1250 kPa	λ_r_ = 1.15 ± 0.1

#### Loading scenarios, boundary conditions and fiber realignment

**Figure 3:**
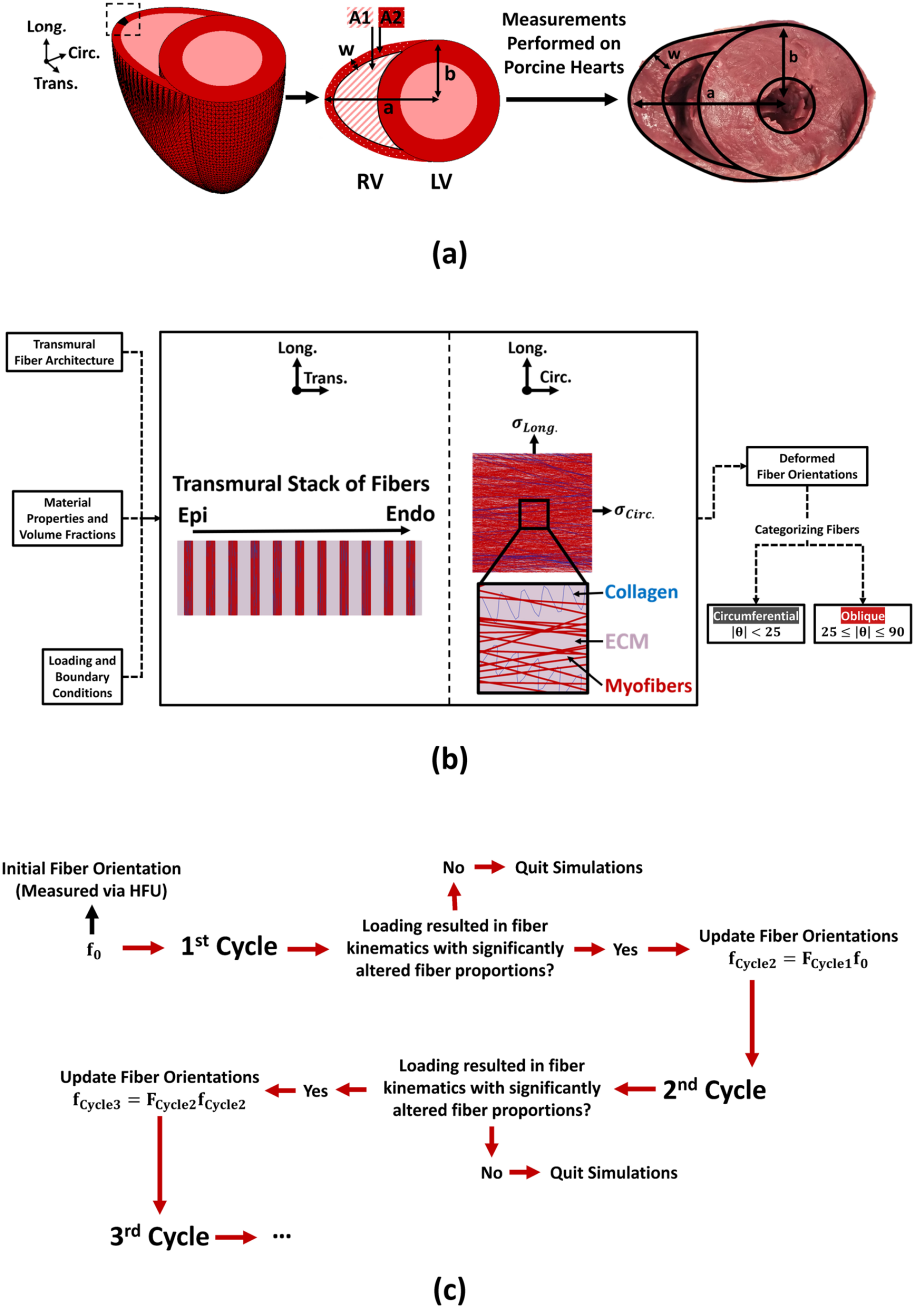
The finite element modeling framework used to study biaxial RV fiber kinematics. (**a**) Loading and boundary condition calculations. In order to keep the computational burden tractable, simulations were performed on a representative volume element (RVE; shown in black in dotted box), and effects of the 3D RV geometry were modeled as loading and boundary conditions based on an extension of the law of Laplace for ellipsoidal geometries. Measurements were performed on porcine hearts to acquire the geometrical parameters required for modeling. (**b**) The RVE in the circumferential-longitudinal and longitudinal-transmural planes, showing the transmural stack of fibers from epi to endocardium and distribution of myofibers, collagen and ECM at each transmural section. Transmural fiber architectures (measured via HFU) and porcine RVFW material properties/volume fractions were used to generate the RVE. The calculated boundary conditions ($\upsigma _{Circumferential}$ and $\upsigma _{Longitudinal}$) were then applied in the circumferential-longitudinal plane to analyze biaxial RV fiber kinematics. Following simulations, fiber orientations of the RVE in the deformed state were categorized into circumferential ($\left|\uptheta \right| < 25$) and oblique ($25 \le \left|\uptheta \right| \le 90$) fibers. (**c**) The remodeling algorithm used for multi-cycle simulations to study the adaptation of RV fibers towards the longitudinal direction in chronic pressure overload. Deformed fiber orientations were analyzed at the end of each cycle. If loading altered the fiber proportions in a statistically significant manner, fiber orientations were updated using the described criteria and simulations were proceeded to the next cycle. In case of no loading-induced alteration in the proportion of fibers, simulations were aborted. *Circ.* circumferential, *Long.* longitudinal, *Trans.* transmural, *RV* right ventricle, *LV* left ventricle, *a* radius of the ellipsoid representing the RV, *b* LV chamber outer radius, *w* RV wall thickness, *A1* area 1, *A2* area 2, *Epi* epicardium, *Endo* endocardium, *ECM* extracellular matrix, *HFU* high-frequency ultrasound.

Similar to previous work^39,45,50^, we employed a two-level modeling approach to keep the computational burden tractable. While simulations were performed on a representative volume element (RVE; 1 mm × 1 mm × RV thickness) of the RVFW incorporating tissue level structural organization (Fig. 3), loading and boundary conditions were based on an extension of the law of Laplace, representing the entire RV approximated by an ellipsoidal geomtery^51,52^ (Fig. 3a):

9 $$ {\text{p}} = \frac{1}{{2\left( {{\text{b}} - {\text{w}}} \right)^{2} }}\left[ {{\upsigma }_{{{\text{Circ}}.}} \left( {{\text{a}}^{2} - \left( {{\text{a}} - {\text{w}}} \right)^{2} } \right) + {\upsigma }_{{{\text{Long}}.}} \left( {\frac{{{\text{b}}^{4} }}{{{\text{a}}^{2} }} - \frac{{\left( {{\text{b}} - {\text{w}}} \right)^{4} }}{{\left( {{\text{a}} - {\text{w}}} \right)^{2} }}} \right)} \right] $$

10 $$ {\text{pA}}_{1} =\upsigma _{{{\text{Long}}.}} {\text{A}}_{2} $$

11 $$ \begin{aligned} {\text{A}}_{1} { } & = { }\frac{{\uppi }}{2}\left[ {\left( {{\text{a}} - {\text{w}}} \right)\left( {{\text{b}} - {\text{w}}} \right) - {\text{b}}^{2} } \right] + {\text{arctan}}\left( {\frac{b - w}{{a - w}}\sqrt {\frac{{\left( {{\text{a}} - {\text{w}}} \right)^{2} - {\text{b}}^{2} }}{{2{\text{bw}} - {\text{w}}^{2} }}} } \right){\text{b}}^{2} - \\ & \quad \ldots { }\left( {{\text{a}} - {\text{w}}} \right)\left( {{\text{b}} - {\text{w}}} \right){\text{arctan}}\left( {\frac{{\left( {{\text{b}} - {\text{w}}} \right)^{2} }}{{\left( {{\text{a}} - {\text{w}}} \right)^{2} }}\sqrt {\frac{{\left( {{\text{a}} - {\text{w}}} \right)^{2} - {\text{b}}^{2} }}{{2{\text{bw}} - {\text{w}}^{2} }}} } \right) \\ \end{aligned} $$

12 $$ {\text{A}}_{2} { } = { }\frac{\uppi }{2}\left[ {{\text{ab}} - {\text{b}}^{2} } \right] - {\text{A}}_{1} $$

Here, p is the internal chamber pressure of the RV (diastolic or systolic pressure), σ_Circ._ and σ_Long_ are the RVFW wall stress (afterload) in the circumferential and longitudinal directions, w is the RV wall thickness, a and b are geometrical measures for biventricular modeling of the left and right ventricles using Laplace-type calculations (Fig. 3a) and A_1_ and A_2_ respectively represent the internal RV chamber area and the RVFW area, when looking at a basal short-axis cross-section of the ventricles (Fig. 3a). Equations 9–12 approximate the RV geometry with an oblate spheroid (semi-axes: a, a, b) while the left ventricle (LV) is approximated with a prolate spheroid (semi-axes: b, b, L; when L represents the LV length). Detailed derivation of these equations have been described elsewhere^51^ and employed to estimate RV wall stress under pressure overload. Myofiber diameter (57.8 ± 28.3 µm) and spacing (35.8 ± 11.2 µm) were acquired form histological measurements (Fig. 2b) to generate the appropriate number of fibers in the RVE (network of 79,263 fibers generated transmurally). Measurements were performed on 3 porcine hearts to obtain the required geometrical measures for modeling (Fig. 3a; a = 64.7 ± 5.8 mm, b = 34.7 ± 2.5 mm, w = 9.8 ± 1.6 mm, and L = 65.0 ± 7.1 mm). Since systolic wall stress reaches peak values early in systole^53^ with minimal dimension change from end-diastole^54^, same RV geometries were assumed to calculate wall stresses at end-diastole and early systole (Tables 2, 3). Calculated wall stresses were then applied to the developed RVE to study the stretch-induced kinematics of RV fibers (Fig. 3b). First, we investigated biaxial kinematics under normotensive loading with healthy RV pressures and material properties and no major remodeling event present in the model (Tables 2, 3). Additionally, an acute pressure overload (APO) scenario was studied to investigate the effects of acute pressure rise on biaxial fiber kinematics. Likewise, effects of chronic pressure overload (CPO) on fiber kinematics was studied under PH pressures^34^ post-remodeling, while RVFW fibrosis^6^, eccentric^34^, and concentric^33^ hypertrophy (RV dilation and increased wall thickness, respectively) are present in the model (Tables 2, 3). Since RV fiber realignment in PH is generally viewed as an end-stage remodeling event^6,8^, this will help analyzing the alterations in fiber kinematics post RV remodeling in CPO. Furthermore, due to the previously reported associations between aging and diminution of the extent of concentric RV hypertrophy in response to pressure overload^54^, as well as lack of increased wall thickness in some animal models of PH^8,55^, another case of CPO loading without concentric hypertrophy was simulated to study the role of increased wall thickness on fiber kinematics in PH (Tables 2, 3). Moreover, a series of simulations were conducted to analyze the effects of RV fibrosis on PH-induced fiber kinematics by parametrically altering the collagen content in our model (1.5, 2 and threefold increase; supplementary Fig. S2). This will help decoupling the observed effects from fibrosis, concentric and eccentric RV hypertrophy under CPO.

**Table 2:** RV hemodynamics and remodeling parameters used for FE simulations.

	RV pressures (mmHg)^34^		End-diastolic volume (mL)^34^	RVFW thickness (mm)^33^	Collagen volume fraction^6,47,48^
	End-diastole	Early-systole			
Normotensive	4.9	31.3	27.7	9.8	4%
Acute pressure overload	14.3	105	27.7	9.8	4%
Chronic pressure overload	14.3	105	34.1	13.4	12%
Chronic pressure overload without concentric hypertrophy	14.3	105	34.1	9.8	12%

**Table 3:** Circumferential and longitudinal wall stresses applied to the representative volume element (RVE) for different loading scenarios.

	End-diastole		Early-systole	
	$\upsigma _{{{\text{Circ}}{.}}}$ (kPa)	$\upsigma _{{{\text{Long}}{.}}}$ (kPa)	$\upsigma _{{{\text{Circ}}{.}}}$ (kPa)	$\upsigma _{{{\text{Long}}{.}}}$ (kPa)
Normotensive	0.62	0.37	3.96	2.37
Acute pressure overload	1.81	1.08	13.30	7.94
Chronic pressure overload	0.87	0.66	6.37	4.81
Chronic pressure overload without concentric hypertrophy	1.53	1.58	11.22	11.64

Simulations were performed under diastolic and early-systolic pressures for a single cycle of loading, in order to analyze the direction and rate of fiber reorientation under different scenarios. Since a single cycle of loading may not result in statistically significant differences in the mean of fiber orientation distributions^56^, similar to previous studies^34,56^, post-deformation fiber orientations were categorized as circumferential ($\left|\uptheta \right| < 25$) and oblique ($25 \le \left|\uptheta \right| \le 90$), to facilitate statistical testing of changes in the proportion of fiber orientations (Fig. 3b). Thresholds for categorizing circumferential/oblique fibers were chosen in a way to include the undeformed fiber orientations ± at least 1 standard deviation of fiber spreads (dispersion) within the circumferential range (supplementary Fig. S3). Therefore, an increase in the proportion of oblique fibers indicates fiber reorientation away from the circumferential direction, as seen in the setting of PH^5,6,8,51^. In case of no statistically significant alteration in the proportion of fiber orientations, simulations were aborted after the first cycle (Fig. 3c). Since, physiologically, fiber remodeling in PH happens over multiple cycles of loading^5,6,8^, when proportions were significantly altered, fiber orientations were updated using the deformation gradient tensor and simulations were proceeded to the next cycle (Fig. 3c):

13 $$ {\text{f}}_{{{\text{n}} + 1}} = {\text{F}}_{{\text{n}}} {\text{f}}_{{\text{n}}} $$

Here, f_n_ is the undeformed orientation of any given fiber at the nth cycle, F_n_ is the deformation gradient tensor for the nth cycle and f_n+1_ represents the undeformed fiber orientation at the start of the n + 1th cycle. This is conceptually similar to previously developed models for stretch-induced fiber remodeling in other cardiovascular soft tissues^22^. In the current exploratory work, simulations were continued up to 4 cycles under CPO for an exploratory assessment of the trends in fiber realignment in PH. FE model post-processing was performed using ParaView^57^ (Sandia National Labs, Kitware Inc, and Los Alamos National Labs).

### Statistical analysis

Data is presented with mean ± standard deviation (SD)/standard error of the mean (SEM), or proportion (%) of fibers post-categorization. A one-way repeated measures ANOVA with Tukey’s post-hoc was used for pairwise comparison of RV helix slope before and after loading and theoretical predictions of affine reorientation. A R^2^ measure was used to evaluate the agreement between experimentally measured uniaxial fiber kinematics and theoretical estimations or FE model predictions. In addition, R^2^ measures were also used to evaluate the goodness of fit between experimentally measured biaxial mechanical properties of the RVFW and FE model predictions.

Chi-squared (χ^2^) tests were performed to evaluate if fiber proportions were dependent on the loading condition. Post-hoc Z-tests with Bonferroni correction were utilized to test the differences across different loading scenarios. Due to integer overflow issues and limitations of the χ^2^ test for very large sample sizes, fiber counts were down sampled by a factor of 10, post categorization. This was chosen as an optimal down sampling rate required to avoid numerical instabilities when working with very large sample sizes (n = 79,263) from our FE model.

Statistical comparisons were performed using the R software package^58^ (R Foundation for Statistical Computing, Vienna, Austria, www.R-project.org). Due to the small sample size in our experimental data and proportion testing (as opposed to tests of the mean) for FE simulations, a more strict measure was chosen for statistical comparisons. For all purposes, *p* < 0.01 (two-sided) was considered statistically significant.

## Results

### HFU imaging

Transmural RV myofiber orientations measured via HFU imaging and histology are compared in Fig. 2b. HFU imaging showed an acceptable agreement with histological staining of fiber orientations both in terms of the dominant orientation and spread of myofibers. Unloaded RV fiber orientations in the basal anterior zone demonstrated an abrupt change^42^ around the sub-epicardial layers (142.4 ± 52.1°; n = 4; 1 specimen used for histological validation + 3 specimens for uniaxial loading experiments), followed by a near-linear change in transmural fiber angles (28.5 ± 9.8°; n = 4). The abrupt sub-epicardial change showed high between-sample variability, ranging from 64.7 to 175.2° (n = 4).

**Figure 4:**
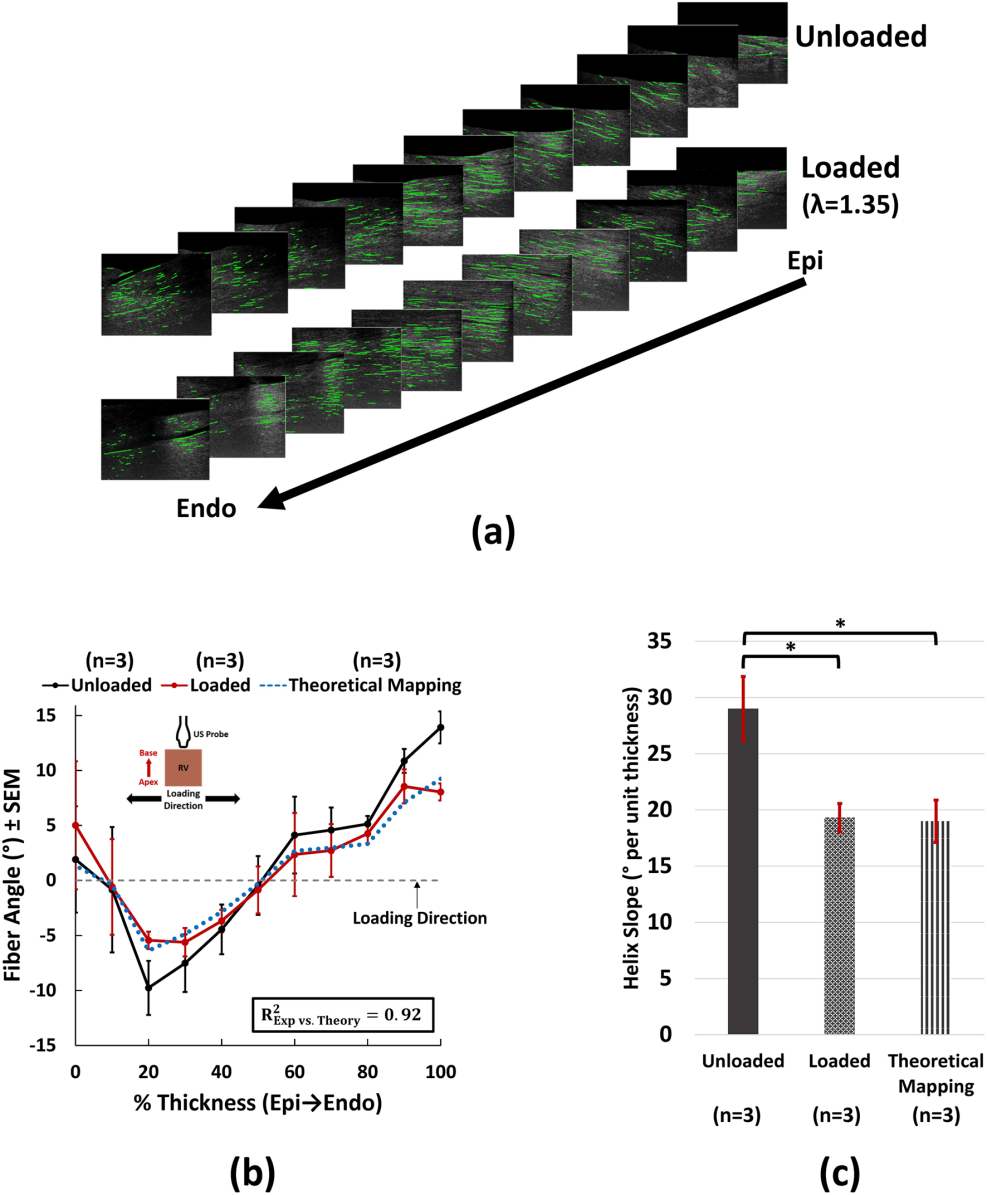
Uniaxial myofiber kinematics under a stretch ratio of λ = 1.35. (**a**) Representative transmural HFU images before and after loading. (**b**) Quantification of fiber angles using the developed framework. Uniaxial loading results in fiber reorientation towards the loading direction (0°). Experimental measurements demonstrate good agreement with theoretical approximations of affine fiber kinematics for an incompressible transversely isotropic solid (R^2^ = 0.92). (**c**) Effects of loading on RVFW helix slope. Loading results in decreased RVFW helix slope, which does not show any statistically significant differences with theoretical affine approximations. Error bars show standard error of the mean (SEM). *Indicates *p* < 0.01. One-way repeated measures ANOVA with post-hoc testing performed using Tukey’s test. *Epi* epicardium, *Endo* endocardium, *US Probe* ultrasound probe, *Exp* experimental, *RV* right ventricle, *RVFW* right ventricular free wall.

**Figure 5:**
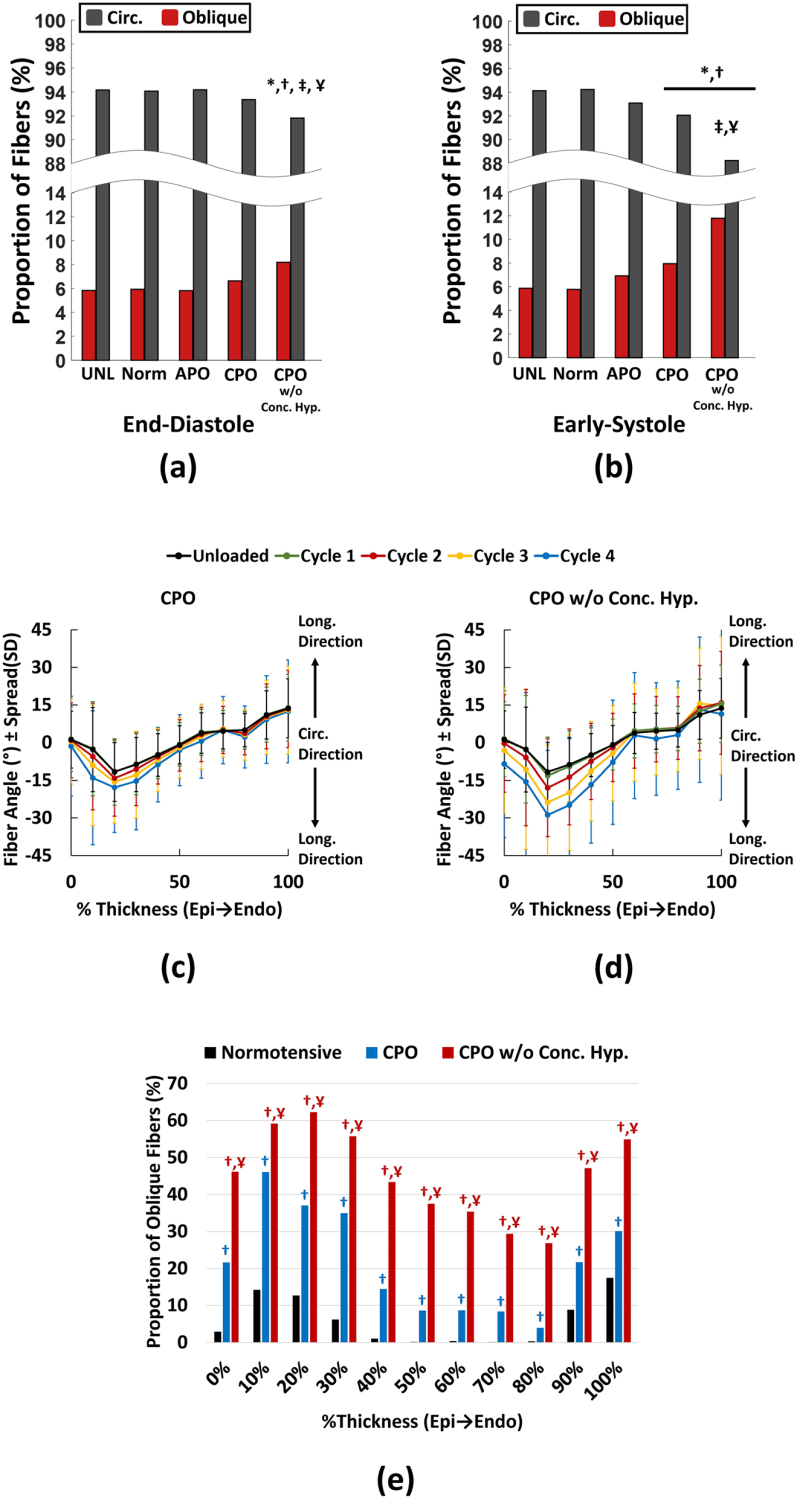
Effects of different loading scenarios on biaxial fiber kinematics of RV myocardium. (**a**) Effects of loading on end-diastolic fiber kinematics, indicating increased proportion of oblique fibers only for the CPO scenario without concentric hypertrophy (single-cycle simulations). (**b**) Effects of loading on early-systolic fiber kinematics, demonstrating elevated proportion of oblique fibers under both CPO scenarios with amplified proportions for CPO without concentric hypertrophy (single-cycle simulations). (**c**) Multi-cycle simulations of RV fiber reorientation towards the longitudinal direction (± 90°) under CPO and (**d**) CPO without concentric RV hypertrophy. Error bars demonstrate fiber spread (standard deviation of the distribution of fiber orientations) at each transmural section. (**e**) Proportion of oblique fibers for the normotensive scenario compared to CPO with and without concentric hypertrophy. Fiber proportions for the CPO cases are plotted following 4 cycles of remodeling simulations, while normotensive loading quits the remodeling algorithm following the first cycle of loading. Both CPO scenarios result in significant increases in the proportion of oblique fibers compared to normotensive loading at all transmural sections (epi to endocardium). Increased wall thickness (CPO vs. CPO w/o Conc. Hyp.) decreases the rate of longitudinal realignment, indicating a potential protective role for concentric hypertrophy against RV fiber reorientation in CPO. *, †, ‡ and Ұ indicate *p* < 0.01 compared to UNL (unloaded), Norm (normotensive), APO (acute pressure overload) and CPO (chronic pressure overload), respectively. Chi-squared tests followed by post-hoc z-tests with Bonferroni corrections. *RV* right ventricle, *Circ.* circumferential, *Long.* longitudinal, *UNL* unloaded, *Norm* normotensive, *APO* acute pressure overload, *CPO* chronic pressure overload, *CPO w/o Conc. Hyp.* chronic pressure overload without concentric hypertrophy, *Epi* epicardium, *Endo* endocardium.

**Figure 6:**
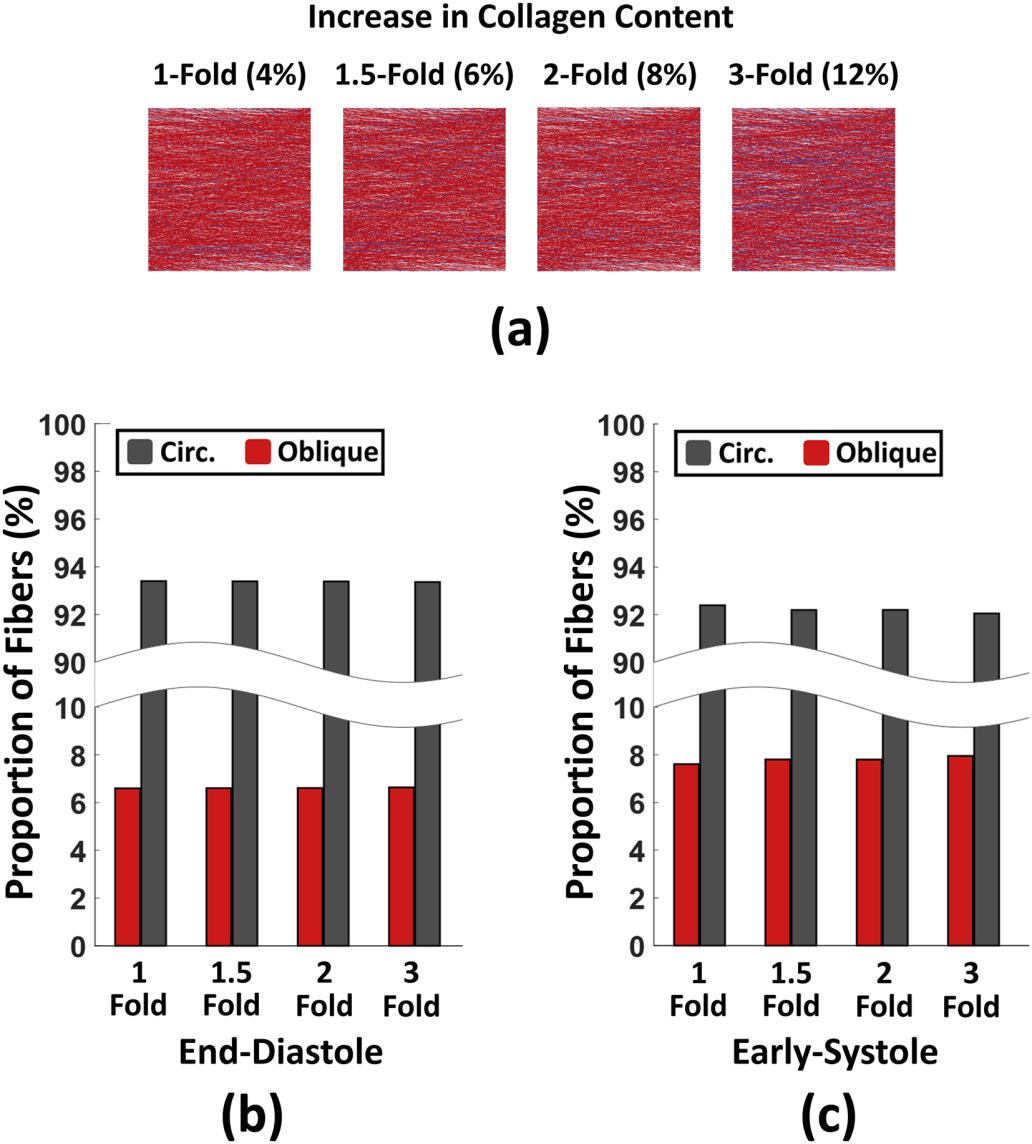
Effects of fibrosis on biaxial RV fiber kinematics under chronic pressure overload with concentric and eccentric hypertrophy. (**a**) Parametric increase in RV collagen content. Red: Myofibers, Blue: Collagen. (**b**) Effects of fibrosis on end-diastolic and (**c**) Early-systolic fiber kinematics. RV fibrosis did not demonstrate any statistically significant alteration in fiber proportions at end-diastole or early-systole. Chi-squared tests. *Circ* circumferential, *RV* right ventricle.

Uniaxial loading (n = 3) resulted in fiber realignment towards the loading direction (Fig. 4a, b), in agreement with theoretical predictions of affine fiber kinematics (R^2^ = 0.92; Fig. 4b). For all kinematics analysis purposes (Figs. 4, 5, 6), fiber orientations are reported in the RV circumferential-longitudinal coordinate system (Fig. 4b). Assuming a counterclockwise transmural rotation for RV fibers^5,6,42^, the abrupt epicardial change is plotted with the shortest map to 0° to better demonstrate fiber realignment towards the loading direction (1.9° and − 0.8° shown for 0% and 10% thickness in Fig. 4b correspond to 180° + 1.9° = 181.9° and 180°-0.8° = 179.2°, respectively). Uniaxial loading resulted in significantly reduced RV helix slope (19.3 ± 2.2° vs. 29.0 ± 5.0° per unit thickness for loaded and unloaded, respectively; *p* < 0.001), while showing no significant differences with theoretical predictions of uniaxial affine fiber kinematics (19.0 ± 3.3° per unit thickness; *p* < 0.001 vs. unloaded and p = 0.983 vs. loaded).

### FE Modeling

The developed structurally-informed FE model matched the mechanical properties of RV myocardium in both longitudinal and circumferential directions (R^2^_Long._ = 0.94, R^2^_Circ._ = 0.99, supplementary Fig. S4), as well as the experimentally measured uniaxial fiber kinematics (R^2^ = 0.89, supplementary Fig. S4). Fiber proportions were calculated based on a total of n = 79,263 fibers generated in the FE model. Categorizing RV fibers in an unloaded state resulted in 94.1% circumferential and 5.9% oblique fibers (proportions calculated by grouping all transmural sections together). Full statistical results of biaxial fiber kinematics in the first cycle of loading are presented in supplementary Tables S1–S2. In response to a single cycle of loading, stretch-induced kinematics under normotensive and APO conditions did not increase the proportion of oblique fibers for either end-diastolic (Fig. 5a; 5.9% for Norm, 5.8% for APO) or early-systolic (Fig. 5b; 5.8% for Norm, 6.9% for APO) pressures. CPO, on the other hand, significantly increased the proportion of oblique fibers under early-systolic pressures by 35%, while showing no significant effects at end-diastole (Fig. 5a, b; 8.0% for CPO at early-systole and 6.6% at end-diastole). For CPO with no concentric RV hypertrophy present in the model, proportion of oblique fibers increased by 101% at early-systole and 40% at end-diastole (Fig. 5a, b; 11.8% for CPO w/o Conc. Hyp. at early-systole and 8.2% at end-diastole). Following the first cycle of loading, multi-cycle remodeling simulations (Fig. 3c) were performed for the CPO scenarios under early-systolic pressures. Simulations demonstrated RV fiber realignment towards the longitudinal direction (Fig. 5c, d), with higher remodeling rates for the case without concentric hypertrophy (Fig. 5d). Categorizing RV fibers at each transmural section after 4 cycles of remodeling, showed a significant increase in the proportion of oblique fibers for both scenarios (Fig. 5e; *p* < 0.001 for CPO or CPO w/o Conc. Hyp. compared to Normotensive at all transmural sections). Concentric hypertrophy resulted in significantly smaller proportions of oblique fibers throughout the RVFW thickness (*p* < 0.001 for CPO vs. CPO w/o Conc. Hyp at all transmural sections). Supplementary Fig. S5 demonstrates a representative shift in fiber distributions with increased proportion of oblique fibers for each loading scenario.

Fibrosis (Fig. 6a) did not show any significant effects on RV fiber kinematics under end-diastolic (Fig. 6b; 6.6%, 6.6%, 6.6% and 6.6%, respectively for 1-, 1.5-, 2-, and 3-fold increase in collagen content) or early-systolic pressures (Fig. 6c; 7.6%, 7.8%, 7.8% and 8%, respectively for 1-, 1.5-, 2-, and 3-fold increase in collagen content).

## Discussion

In this work, we aimed to evaluate the stretch-induced fiber kinematics of RV myocardium under different loading scenarios. The primary findings of this study were: (1) HFU imaging demonstrated a strong potential for quantifying full-thickness transmural orientations of RV myofibers in large animal models; (2) Uniaxial loading resulted in fiber realignment towards the loading direction, in agreement with theoretical predictions based on affine fiber kinematics; (3) FE modeling suggested that a combination of chronic pressure overload with RVFW fibrosis and concentric and eccentric hypertrophy (increased wall thickness and chamber dilation, respectively), but not solely an acute rise in RV pressures, may result in kinematic shift of RV fibers away from the circumferential direction; (4) Computational exploratory assessments revealed a potential protective role for concentric hypertrophy against longitudinal fiber realignment in PH, while demonstrating that eccentric hypertrophy may stimulate fiber reorientation towards the longitudinal direction.

HFU imaging effectively characterized the transmural orientation distribution (dominant orientation and fiber spread) of myofibers in porcine RV myocardium (Fig. 2). Difficulties were observed in delineation of the endocardial surface via HFU imaging, mainly due to the trabeculations at endocardium, as also reported in previous studies^59^. This resulted in differences between the HFU and histologically measured fiber spreads at endocardium, while dominant fiber orientations were accurately characterized (Fig. 2b). As previously reported in porcine models of RV structure^42^, an abrupt change in fiber angles with high between-sample variability was observed near the sub-epicardial layers. This was followed by a near-linear change in orientations towards the endocardium (Fig. 2b). The observed transmural variation in fiber angles (epi → endo) in the basal anterior zone (28.5 ± 9.8°) was smaller than previously reported values, potentially due to the body size and procurement source of tissues used in this study (farmed animals with ≈ 80–120 kg body weight) compared to younger animals used in previous work (≈ 5–20 kg body weight^12,16^). Decreased transmural variation in fiber orientations with increased body size has also been reported in prior investigations^60^.

As expected, uniaxial loading of RV myocardium resulted in realignment of fibers towards the loading direction with decreased transmural variation (Fig. 4). While not representing a physiological loading experienced by the RVFW in vivo, uniaxial loading can provide fundamental insights into the kinematic response of RV fibers, which remains poorly understood. Since fiber reorientation in PH happens over multiple cycles of growth and remodeling^8^, as anticipated, we did not observe dramatic changes in fiber angles under a single cycle of passive uniaxial loading. However, RV myofibers demonstrated a strong agreement with theoretical predictions of uniaxial affine kinematics (Fig. 4b, c), thus indicating affine assumptions can effectively approximate myofiber realignment in the RVFW. To the best of our knowledge, this is the first report on quantitative HFU assessment of full-thickness RV fiber kinematics.

FE simulations revealed the role of stretch-induced deformations on longitudinal kinematics of RV fibers in PH. In an unloaded state, circumferential fibers dominated the fiber proportions in the RVFW. This is in general agreement with previous studies on a porcine model^16^, indicating the basal anterior zone of RVFW as one of the most circumferentially aligned regions. Small, but significant alterations in fiber proportions were noted after a single cycle of loading under pressure overload conditions. The observed changes in fiber angles in the first cycle of loading are in general agreement with previously reported values^17^ (Fig. 5a, b). As anticipated, normotensive loading did not demonstrate any significant effects on the proportions of RV fibers (Fig. 5a, b). Even though an acute pressure rise imposes the largest wall stresses on the RVFW (Table 3), surprisingly, this did not significantly alter the proportion of RV fibers. This is potentially due to the importance of the ratio (and not just absolute values) of the biomechanical stimuli (strain) in fiber reorientation^22^. While RV stresses are highest during APO, this may not result in deformed configurations required for stimulating longitudinal fiber kinematics (Supplementary Fig. S1). This is mainly a combined effect from hemodynamic pressures, RV geometry and RVFW biomechanical properties in APO that lead to deformation states where ratio of longitudinal:circumferential strains does not stimulate longitudinal fiber reorientation. In contrast, CPO with a combination of remodeling events (Table 2; increased wall thickness, RV dilation, and fibrosis) significantly increased the proportion of oblique fibers (Fig. 5), indicating fiber remodeling away from the circumferential direction. This is in agreement with prior studies, identifying fiber realignment as an end-stage event in PH^6,8^. Elimination of increased wall thickness from our FE models amplified fiber realignment towards the longitudinal direction (Fig. 5c, e). This suggests a potential protective role for increased wall thickness against fiber reorientation in PH. Animal models of PH without concentric hypertrophy^8^ have demonstrated similar levels of fiber remodeling under significantly lower RV pressures compared to those developing concentric hypertrophy^5,6^. A potential explanation to be further explored in future studies is simultaneous reduction of RV wall stress and alterations in the ratio of longitudinal:circumferential strains via increased wall thickness in PH.

Parametric studies on the level of RV fibrosis did not show any effects on fiber proportions under early-systolic or end-diastolic pressures (Fig. 6). Between the three remodeling events studied here (increased wall thickness, dilation, and fibrosis), this leaves RV dilation as a potential contributor to the kinematic shift of RV fibers towards the longitudinal direction in PH. Previous experimental/computational studies have shown fiber realignment in PH to be accompanied by progressive RV dilation^8,12^. Growth and remodeling analysis of RV biomechanics in PH revealed that longitudinal fiber realignment, accompanied by dilation, may result in impaired RV function and reduced ejection fractions^8^. Our computational modeling results suggest another pathway for RV dilation to modulate longitudinal fiber remodeling via altered RVFW wall stress and stretch ratios, in addition to the previously addressed dilation-induced fiber kinematics due to volumetric growth^8^. A potential mechanism to be investigated in future work is alteration in the biomechanical stimuli (ratio and magnitude of stress/strains) in the myofiber niche due to progressive RV dilation. It is also worth noting that while our results demonstrate that RV dilation may induce a “kinematic shift” in myofiber orientations, progression of a kinematic shift to fiber realignment may require multiple cycles of RVFW growth and remodeling. Further long-term longitudinal growth and remodeling studies, looking at the time course of progression of different remodeling events in PH, can provide fundamental insights into the cause-consequence relations between myofiber realignment and various RV remodeling events.

There are limitations to the experimental and FE modeling techniques used in the current work. HFU images were acquired under uniaxial loading, as it is currently not feasible under biaxial loading due to the requirement of imaging normal to the transmural axis (parallel to the RVFW) which necessitates at least one side of the RVFW specimen to be free for imaging. Ongoing work focuses on development of improved algorithms using spatial coherence maps to facilitate 3D imaging parallel to the RV transmural axis. While demonstrating strong statistics and minimal variabilities, the small sample size (n = 3) used for our exploratory experimental analysis remains as a limitation of the current study. Although our theoretical models provide insights into the stretch-induced fiber kinematics of RV myocardium under biaxial loading, lack of an experimental control group is a limitation of the current work and future experimental studies are needed to confirm the generated hypotheses via FE modeling. Moreover, our study focused on analyzing myofiber kinematics in the basal anterior zone of the RVFW, mainly due to the feasibility of acquiring theoretical wall stress approximations for this region (Eqs. 9–12). Future work will explore the spatial heterogeneities in RV myofiber kinematics using improved HFU imaging algorithms, coupled with 3D structurally-informed organ-level FE models for quantification of wall stress in other regions of the RVFW. Furthermore, as described in Fig. 1, the deformed state of RVFW during early-systole (IVC) includes a stretch-driven deformation in addition to a myofiber contraction mode. Our FE models did not include the contraction mode (myofiber shortening), mainly due to the computational complexity of modeling contraction on an RVE without representing the 3D RV geometry. However, systolic myofiber shortening has been shown to result in deformations with ratio of longitudinal:circumferential strains close to 1 (ratio of global longitudinal to circumferential strains ranging from 0.99 to 1.04 for normotensive subjects, as well as subjects with hypertrophic or dilated cardiomyopathy^61^). Therefore, while this affects the magnitude of the strains calculated in our models, it is less likely to affect the ratio of longitudinal:circumferential strains (mainly responsible for stimulating fiber rotations; supplementary Fig. S1) in a way to obstruct the observed trends in longitudinal realignment of RV fiber. Additionally, end-stage PH has been shown to reduce systolic myofiber shortening^18^, which makes the contraction mode less likely to mechanistically stimulate transmural fiber reorientation in PH. Our results only explain a specific stretch-driven aspect of RV fiber realignment and future work will focus on development of 3D organ-level FE simulations to effectively model the fiber kinematics at early-systole considering both deformation modes. Moreover, even though the underlying mechanisms of fiber reorientation in PH remains largely unexplored, reorientation due to altered fiber kinematics and biomechanical stimuli in the myofiber niche^8,21,22^ is only one of the several potential mechanisms responsible for this remodeling event. Other mechanisms such as degradation of circumferentially aligned fibers accompanied by sarcomerogenesis of longitudinal fibers (preferential hypertrophy)^8^ need to be further investigated in future fiber-level studies.

In conclusion, we employed HFU imaging and structurally-informed computational models for an exploratory study on the stretch-induced kinematics of RV fibers under different loading scenarios. The developed imaging framework provides a noninvasive modality to characterize the transmural fiber kinematics of RV myocardium. Simulations suggest a potential protective role for concentric RV hypertrophy (increased wall thickness) against fiber reorientation. On the other hand, accounting for eccentric hypertrophy (RV dilation) in FE models resulted in kinematic shift of fibers towards the longitudinal direction, due to altered stretch ratios in the RVFW. While this helps better understanding the role different remodeling events and biomechanical forces in transmural realignment of RV fibers in PH, future experimentations are warranted to test the model-generated hypotheses.

## Supplementary Information

Supplementary tables.

## Data Availability

The data generated/analyzed during this study are available from the corresponding author on reasonable request.
