# Supplementary material for: An exploratory assessment of stretch-induced transmural myocardial fiber kinematics in right ventricular pressure overload
Source: Sci Rep. 2021 Feb 11;11:3587. doi: 10.1038/s41598-021-83154-8 (PMC7878470; doi:10.1038/s41598-021-83154-8)
Supplement: Supplementary file 1 — Supplementary tables. [file 41598_2021_83154_MOESM1_ESM.pdf]

Supplementary Materials:

An Exploratory Assessment of Stretch-Induced Transmural Myocardial Fiber Kinematics in Right Ventricular Pressure Overload

Danial Sharifi Kia, PhD[1], Ronald Fortunato, BS[2], Spandan Maiti, PhD[1,2], Marc A. Simon, MD, MSc[1,3,4,5,6] Kang Kim, PhD[1,2,3,4,5,6,7*]

[1]Department of Bioengineering, University of Pittsburgh, Pittsburgh, PA
[2]Department of Mechanical Engineering and Materials Science, University of Pittsburgh, Pittsburgh, PA
[3]Division of Cardiology, School of Medicine, University of Pittsburgh, Pittsburgh, PA
[4]Heart and Vascular Institute, University of Pittsburgh Medical Center (UPMC), Pittsburgh, PA
[5]Pittsburgh Heart, Lung, Blood and Vascular Medicine Institute, University of Pittsburgh and University of Pittsburgh Medical Center (UPMC), Pittsburgh, PA
[6]McGowan Institute for Regenerative Medicine, University of Pittsburgh, Pittsburgh, PA
[7]Center for Ultrasound Molecular Imaging and Therapeutics, University of Pittsburgh, Pittsburgh, PA

***Address correspondence to:**
Kang Kim, PhD
Associate Professor of Medicine,
Division of Cardiology, Department of Medicine,
University of Pittsburgh School of Medicine,
623A Scaife Hall, 3550 Terrace Street, Pittsburgh, PA, 15213
Email: kangkim@upmc.edu
Tel: (412) 624-5092
Fax: (412) 624-2264

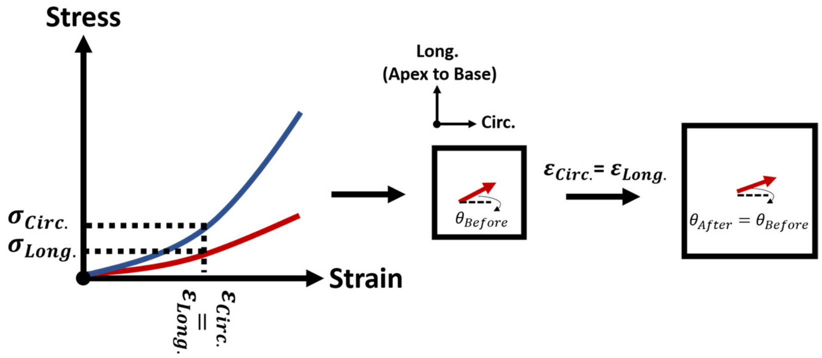

(a)

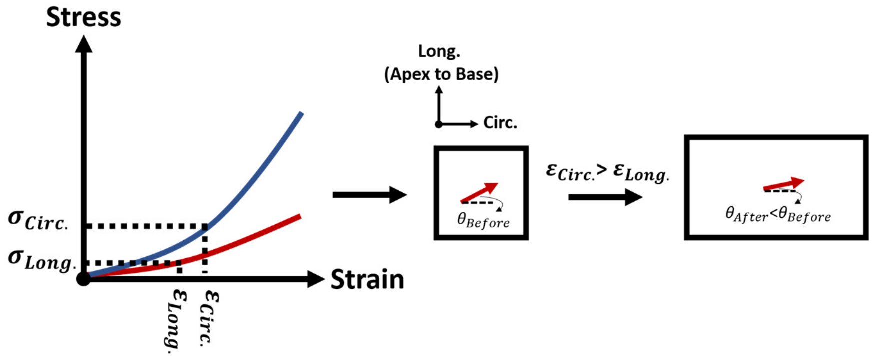

(b)

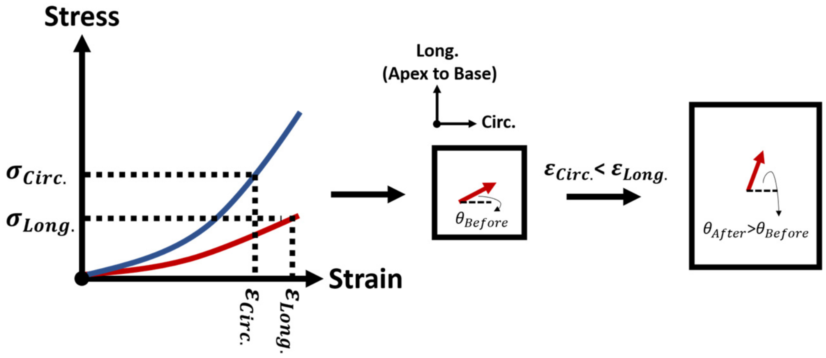

(c)

Supplementary Figure S1: Conceptual demonstration of the effects of biaxial loading conditions on the deformation state and fiber kinematics of RV myocardium. Hypothetical loading scenarios resulting in **(a)** equibiaxial strain with no fiber rotations as well as scenarios with kinematic shift of fibers towards the **(b)** circumferential and **(c)** longitudinal directions. Material nonlinearity and anisotropy of RV myocardium results in different fiber kinematics at different stress levels. While some scenarios may result in equibiaxial strain conditions with no fiber kinematics, others may lead to $\varepsilon_{Circ.} > \varepsilon_{Long.}$ or $\varepsilon_{Circ.} < \varepsilon_{Long.}$, resulting in clockwise or counterclockwise fiber kinematics, respectively.
Circ: Circumferential; Long.: Longitudinal; RV: Right ventricle.

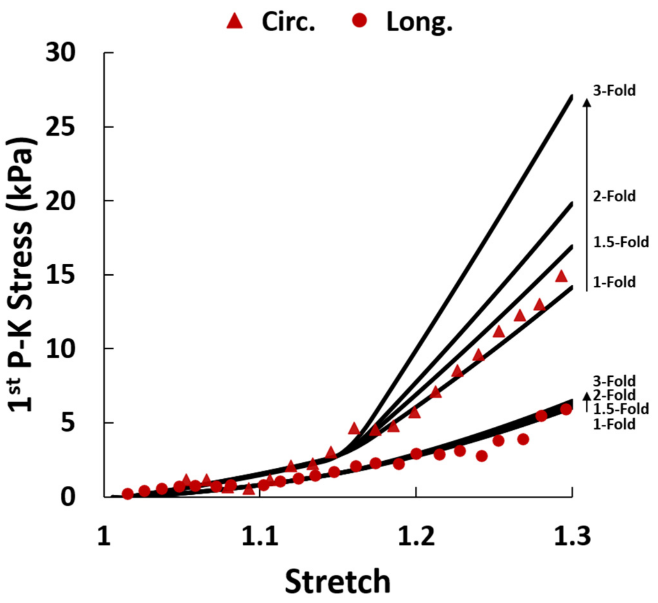

Supplementary Figure S2: Effects of parametric increase in RVFW collagen content on the biaxial mechanical properties of RV myocardium. Experimental data (shown in red) obtained from the literature[1]. Material properties of the structurally-informed finite element (FE) model shown with black lines.

Circ.: Circumferential; Long.: Longitudinal; 1st P-K Stress: 1st Piola-Kirchhoff stress; RV: Right ventricle; RVFW: Right ventricular free wall.

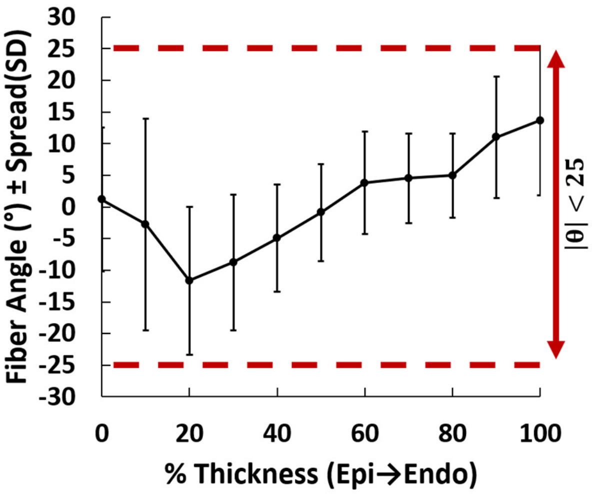

(a)

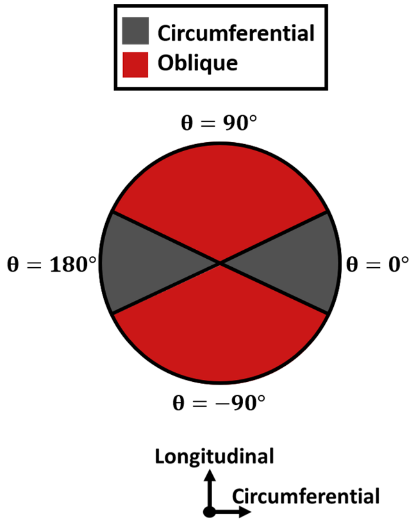

(b)

Supplementary Figure S3: Rationale for the threshold used for categorizing circumferential and oblique fibers. **(a)** Fiber angle threshold used for categorizing circumferential fibers ($|\theta| < 25$). **(b)** Graphical demonstration of circumferential and oblique fibers in a circular plane. Deformed RV fiber orientations were grouped into circumferential ($|\theta| < 25$) and oblique ($25 \leq |\theta| \leq 90$) categories, following finite element simulations. The threshold for circumferential fibers was chosen in a way to include the initial HFU-measured fiber angles and at least 1 standard deviation of the fiber spread at each transmural section. Fibers outside of this threshold were labeled as "oblique". An increase in the proportion of oblique fibers indicates fiber reorientation away from the circumferential direction, towards the longitudinal direction (±90°). Error bars demonstrate the fiber spread (pooled standard deviation of the distribution of fiber orientations measured via HFU imaging on n=3 specimens) at each transmural section.

Epi: Epicardium; Endo: Endocardium; RV: Right ventricle.

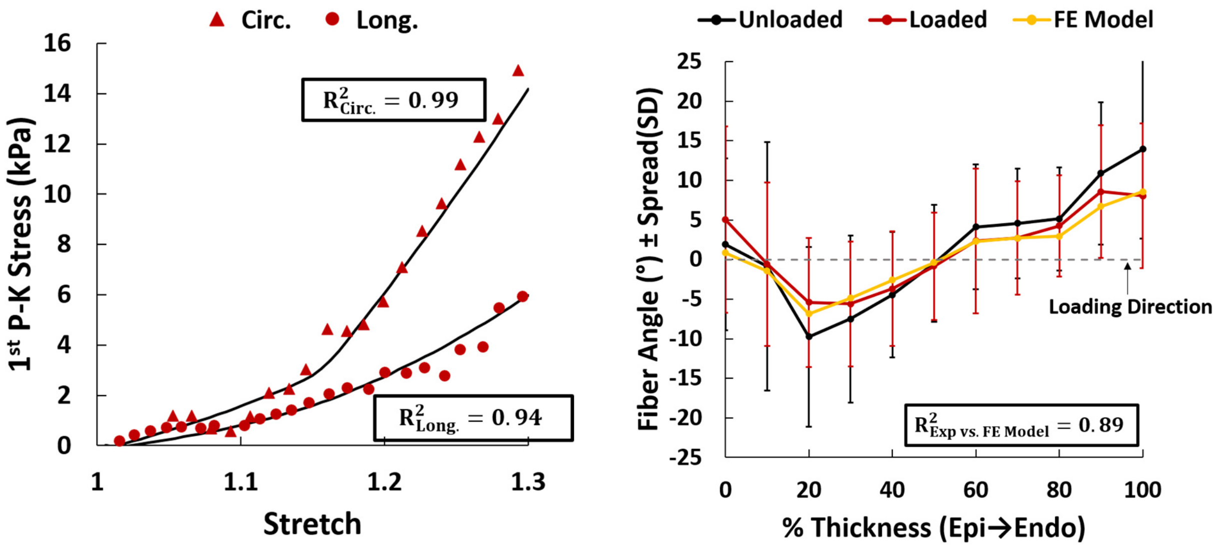

(a) **(b)**

Supplementary Figure S4: **(a)** Quality of fit of the structurally-informed finite element (FE) model to previously reported[1] biaxial material properties of porcine RV myocardium. **(b)** FE model predictions vs. experimentally measured uniaxial RV fiber kinematics. Error bars demonstrate the fiber spread at each transmural section.

Circ.: Circumferential; Long.: Longitudinal; 1st P-K Stress: 1st Piola-Kirchhoff stress; RV: Right ventricle; Exp: Experimental; Epi: Epicardium; Endo: Endocardium.

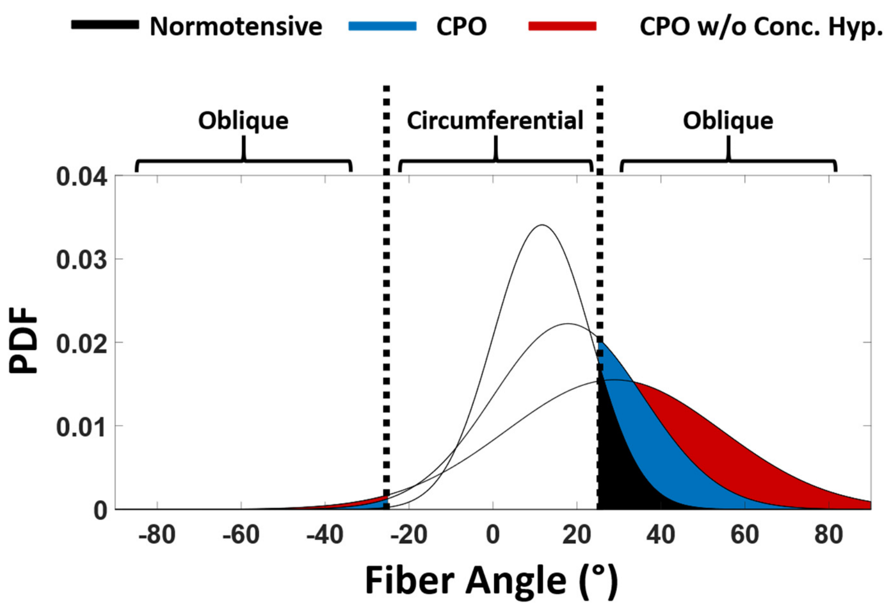

Supplementary Figure S5: Representative fiber orientation distributions for multi-cycle remodeling simulations under chronic pressure overload (CPO) (Fig. 5c-e), demonstrating a shift towards oblique fibers for both CPO scenarios. Shaded area under the curves demonstrate the oblique fibers. Both CPO scenarios result in remodeling towards the longitudinal direction (±90°) with increased proportion of oblique fibers. As demonstrated, in addition to an increase in the proportion of oblique fiber, dominant orientation of the distributions (mean/median of a normal distribution) also shifts towards the longitudinal direction (±90°).

CPO: Chronic pressure overload, CPO w/o Conc. Hyp.: Chronic pressure overload without concentric RV hypertrophy, PDF: Probability density function.

Supplementary Table S1: p values obtained for pairwise comparison of different loading scenarios at end-diastole (Fig. 5a)

	**Norm**	**APO**	**CPO**	**CPO w/o Conc. Hyp.**
**UNL**	1.0	1.0	0.4582	<0.001
**Norm**	-	1.0	0.8105	<0.001
**APO**	-	-	0.4137	<0.001
**CPO**	-	-	-	0.0034

Supplementary Table S2: p values obtained for pairwise comparison of different loading scenarios at early-systole (Fig. 5b)

	**Norm**	**APO**	**CPO**	**CPO w/o Conc. Hyp.**
**UNL**	1.0	0.0968	<0.001	<0.001
**Norm**	-	0.0446	<0.001	<0.001
**APO**	-	-	0.1801	<0.001
**CPO**	-	-	-	<0.001

References

1. Nemavhola, F. Biaxial quantification of passive porcine myocardium elastic properties by region. *Eng. Solid Mech.* **5**, 155–166 (2017).
